# Using genetic variations to reveal the complex relationships between vegetarianism and well-being, depressive symptoms and neuroticism

**DOI:** 10.3389/fnut.2024.1389000

**Published:** 2024-10-15

**Authors:** Ke Chen, Yuan Wen, Zhendi Shu

**Affiliations:** ^1^Department of Rehabilitation Medicine, Rehabilitation Medicine Center, The Second Affiliated Hospital and Yuying Children's Hospital of Wenzhou Medical University, Wenzhou, China; ^2^School of Ophthalmology and Optometry, Wenzhou Medical University, Wenzhou, China; ^3^Department of Critical Care Medicine, Wuhan Jinyintan Hospital, Tongji Medical College of Huazhong University of Science and Technology, Wuhan, China

**Keywords:** vegetarianism, depressive symptoms, neuroticism, subjective well-being, mental health

## Abstract

**Background:**

The relationship between vegetarianism and mental well-being remains a debated topic in traditional observational studies. Recent studies have revealed the genetic factors in vegetarianism. We aimed to use genetic variations to explore the potential causal relationships between vegetarianism and mental well-being, offering insights from a new perspective.

**Methods:**

We conducted the inverse variance weighted approach as the primary analysis to explore the bidirectional genetic associations between vegetarianism (*N* = 442,589) and depressive symptoms (*N* = 180,866), neuroticism (*N* = 170,910), and subjective well-being (*N* = 298,420). The analysis used the summary data from the largest genome-wide association studies (GWAS). We also performed sensitivity analyses to ensure the robustness of the findings, accounting for potential heterogeneity and pleiotropy.

**Results:**

Genetically predicted vegetarianism showed positive causal relationships with depressive symptoms (odds ratio [OR], 3.26; 95% confidence interval [CI], 1.03–10.31; *p* = 0.044) and neuroticism (OR, 6.72; 95% CI, 2.29–19.74; *p* = 5.31 × 10^−4^), as well as a negative causal relationship with subjective well-being (OR, 0.20; 95% CI, 0.05–0.77; *p* = 0.019). Additionally, depressive symptoms were found to have a causal influence on vegetarianism (OR, 1.01; 95% CI, 1.00–1.02; *p* = 6.87 × 10^−3^). No significant heterogeneity or pleiotropy was detected.

**Conclusion:**

Vegetarianism is causally correlated with negative mental well-being, reflected in an increased risk of depressive symptoms and neuroticism, as well as lower subjective well-being. Further research should explore the underlying mechanisms in broader populations.

## 1 Introduction

Depression affects millions of people worldwide, significantly reduces quality of life, and correlates with disease burden and mortality (1, 2). It is the most common psychiatric disorder among those who die by suicide (3). However, current treatments like medication and psychological interventions frequently prove inadequate, with depressive symptoms that may relapse (4). Additionally, drug therapy has significant side effects (5), therefore, lifestyle medicine (e.g., exercise, diet, and sleep) has become an area of interest (6, 7).

Neuroticism, one of the “big five” personality traits, is characterized by often experiencing negative emotions such as anxiety, feelings of guilt, loneliness, and fear (8, 9). Depressive symptoms and neuroticism share common features like anxiety and other negative emotions, and neuroticism is a risk factor for depression (9). In contrast, subjective well-being (SWB) involves happiness, life satisfaction, and positive affect, reflecting a good life (10). Higher levels of SWB are often associated with better physical health, longer lifespan, improved social relationships, greater work performance, and enhanced creativity (11).

The impact of diet on mental health and emotions has received increasing attention from researchers. The studies suggested that lifestyle medicine, including dietary adjustments, may offer effective preventive and treatment approaches for depressive symptoms (6). Vegetarianism, commonly defined as a dietary pattern that restricts meat, meat-derived foods, and sometimes other animal-derived products, is growing in popularity worldwide (12). The common motivations include religion, culture, the concerns for the environment, animals, and health (13). Given its unique dietary restrictions, understanding its impact on mental health is crucial. Previous studies have reported conflicting findings on the relationship between vegetarianism and well-being: some found that vegetarians experienced poor well-being (14, 15), while others indicated that vegetarians tended to have better well-being compared to omnivores (16, 17). These differences may be due to different levels of restrictions on animal products, the study population characteristics, and the duration of adherence to a vegetarian diet (18). Moreover, the causal relationships between vegetarianism and depressive symptoms, neuroticism, and SWB remain unclear.

Mendelian randomization (MR) is an analytic approach that uses genetic variants to explore the causal relationship between a potential risk factor and an outcome (19). Genetic variants are assorted naturally and randomly during meiosis, yielding a random population distribution (20). Since gene mutations and random allocation occur before phenotype develops, and genetic variants are unchanged through a lifetime, MR design can reduce confounding factors and reverse causality bias in observational studies (20–22). In this study, we used depressive symptoms, neuroticism, and SWB as three psychological states, conducting bidirectional two-sample MR to evaluate the causal relationships between vegetarianism and these three phenotypes. Our aim was to evaluate the impact of vegetarianism on mental health and offer assistance for supporting vegetarians’ mental well-being.

## 2 Methods

### 2.1 Study design

**Figure 1:**
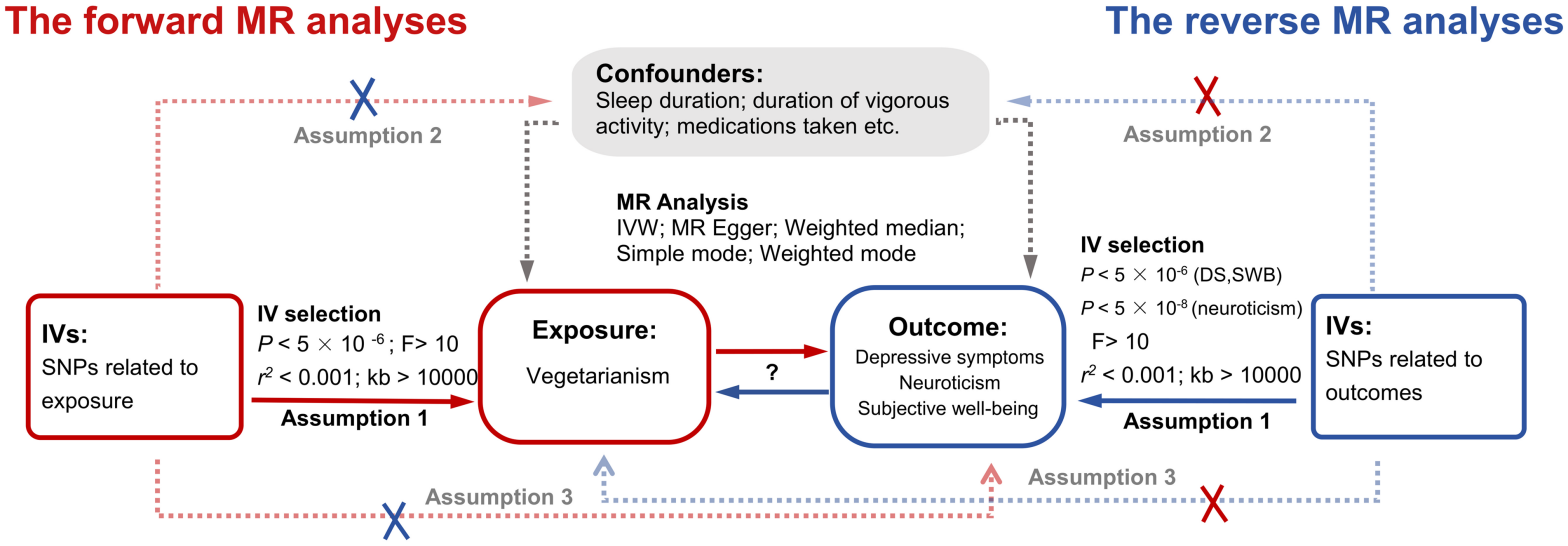
The study design of this bidirectional two-sample MR analysis. Red lines indicate the forward MR analyses (using vegetarianism as exposure and depressive symptoms, neuroticism, and SWB as three outcomes). Blue lines indicate the reverse MR analyses (using depressive symptoms, neuroticism, and SWB as three exposures and vegetarianism as outcomes). IV, instrumental variable; MR, Mendelian randomization; IVW, Inverse variance weighted; SNPs, Single Nucleotide Polymorphisms; DS, depressive symptoms; SWB, subjective well-being.

We used bidirectional two-sample MR to investigate the causal relationships between vegetarianism and depressive symptoms, neuroticism, and SWB. Our study adhered to the latest guidelines for performing MR investigations (23) and referred to several published MR studies. Data were sourced from the summary studies for genome-wide association studies (GWAS). Single nucleotide polymorphisms (SNPs) served as the instrumental variables (IVs) to explore the causal relationships between the exposure and outcome. The study followed the three major assumptions of MR (22): (1) Correlation assumption: the genetic variants are strongly correlated with the exposure; (2) Independence assumption: the genetic variants are not related to potential confounding factors; (3) Exclusivity assumption: the genetic variants influence the outcomes only through the exposure. The detailed research design is shown in Figure 1. Ethical approval was not required for this study, given its exclusive reliance on accessible public GWAS summary statistics.

### 2.2 GWAS data on vegetarianism

The data on vegetarianism was derived from a GWAS in 2022, which used genetic variants to evaluate the association between food intake and health outcomes (24). The research sample of vegetarianism was from UK Biobank, with a sample size of 442,589. The UK Biobank involved 500,000 adults aged 40–69 at baseline across 22 assessment centers in the UK (25). The dietary intake in the UK Biobank was assessed using a touchscreen dietary frequency questionnaire, which included questions about the frequency of consumption of specific foods and beverages over the previous year (24, 25).

The phenotype modeling for vegetarians was based on responses to the following questions (24): “How old were you when you last ate any kind of meat?” and “How often do you eat beef, poultry (chicken, turkey, or other poultry), pork, or lamb/mutton?.” Responses such as “prefer not to answer” and “do not know” were excluded from the analysis. For a detailed description of phenotype modeling, refer to the research by Pirastu et al. (24). Detailed information on GWAS data is presented in Table 1.

**Table 1:** The information and source of GWAS data.

Traits	Data source	Sample size	Population	Consortium	Link
Vegetarianism	Pirastu et al. (24)	442,589	European	NA	https://gwas.mrcieu.ac.uk/datasets/ebi-a-GCST90096927/
SWB	Okbay et al. (8)	298,420	European	SSGAC	https://thessgac.com
Neuroticism		170,910	European	SSGAC	
DS		180,866	European	SSGAC	

SWB, subjective well-being; DS, depressive symptoms; SNPs, Single Nucleotide Polymorphisms; SSGAC, Social Science Genetic Association Consortium.

### 2.3 GWAS data on subjective well-being, depressive symptoms, and neuroticism

The GWAS data of these three traits came from a study in 2016 (8). The detailed description of phenotype modeling refers to the research by Okbay et al. (8). The detailed information on GWAS data is presented in Table 1.

For SWB (*N* = 298,420), the dataset included 59 cohorts. The phenotype measures included life satisfaction, positive affect, or, in some cohorts, a combination of both (8). For depressive symptoms (*N* = 180,866), there were three sources of data: Psychiatric Genomics Consortium (PGC) (Ncases = 9,240, Ncontrols = 9,519) (26), Genetic Epidemiology Research on Aging (GERA) (Ncases = 7,231, Ncontrols = 49,316), and UK Biobank data (UKB) (*N* = 105,739) (25). Both GERA and PGC provided case–control data on major depressive disorder. In the UKB (*N* = 105,739), the phenotype of depressive symptoms was based on participant responses to two questions regarding the frequency with which respondents experienced feelings of unenthusiasm/disinterest and depression/hopelessness in the previous 2 weeks (8). For neuroticism (*N* = 170,910), data came from the Genetics of Personality Consortium (GPC) (*N* = 63,661) (27) and UKB (*N* = 107,245) (25). The GPC harmonized the different neuroticism batteries, while the UKB used the respondent’s score on a 12-item version of the Eysenck Personality Inventory Neuroticism scale for its measure (8).

### 2.4 Instrument variables selection

We conducted a series of analyses and selections to determine qualified SNPs as our IVs for MR analysis. Based on the correlation assumption, we selected SNPs demonstrating strong genetic correlations with exposure (*p* < 5 × 10^−8^) and calculated their F-statistic to avoid weak instrumental bias (*F* > 10). The calculation formula is as follows (28, 29):


$$F={\left(\frac{\beta }{SE}\right)}^{2}$$

where *β* represents the SNP-exposure association estimate, and SE is the standard error. When exposures were vegetarianism, SWB, and depressive symptoms, the number of SNPs meeting *p* < 5 × 10^−8^ was fewer than three; therefore, we used SNPs with *p* < 5 × 10^−6^ to ensure a sufficient number for MR analysis, according to previous MR studies (30). We excluded the SNPs in linkage disequilibrium (LD) (*r^2^* > 0.001 and clump window <10,000 kb) using the PLINK clumping method (31, 32). We removed SNPs strongly correlated with confounding factors or outcomes to adhere to the exclusivity and independence assumptions. We also aligned and removed palindrome structures and incompatible SNPs to harmonize the exposures and outcomes (33). For consistency, only SNPs available for all examined traits were used as IVs, and proxies were not used to replace those missing in outcome data.

### 2.5 MR analysis

We used “TwoSampleMR” “LdlinkR” “forestplot” and “MRPRESSO” packages in R statistical software (version 4.3.3, the R Foundation for Statistical Computing, Vienna, Austria^1^). The fixed effect inverse variance weighted (IVW) method served as the primary MR analysis because of its strict requirement for all SNPs to be effective (34, 35). Supplementary methods, including MR-Egger, weighted median (WM), simple mode, and weighted mode, were used to validate the results. The intercept of MR-Egger reflected the pleiotropy of SNPs, and under the weaker assumption (Instrument Strength Independent of Direct Effect assumption, InSIDE assumption), the slope of MR-Egger regression provided a consistent causal effect estimate (36). The WM method calculated causal effects even if less than 50% of the weight of SNPs was invalid (37). We visualized MR analysis results using forest plots and scatter plots.

We utilized Cochran’s Q test to evaluate the heterogeneity of SNPs (34). Then, we utilized MR-Egger interpret analysis, MR-Pleiotropy RESidual Sum and Outlier (MR-PRESSO) analysis, and “leave-one-out” analysis to detect the pleiotropy of SNPs. The “leave-one-out” analysis identified if a single SNP significantly affected the overall MR estimated effect. The MR-PRESSO global test checked for horizontal pleiotropy, and the MR-PRESSO outlier test was used to eliminate outlier and pleiotropic SNPs (38).

Considering our study had multiple exposures or outcomes, we applied a Bonferroni correction to adjust the significance levels of the *p*-value in forward and reverse MR analyses, respectively (39). A *p*-value < 0.0167 (calculated as *p* < 0.05/3) was considered statistically significant, while a *p*-value > 0.0167 and < 0.05 was regarded as suggestive evidence.

### 2.6 Bias and type I error of sample overlap

We used online programs^2^ to estimate the potential bias and type I error due to sample overlap, ensuring the integrity of our results (40).

### 2.7 Statistical power

We used the programs at a website^3^ to calculate the statistical power in evaluating the causality between vegetarianism and depressive symptoms, neuroticism, and SWB (41). Statistical power exceeding 80% is considered indicative of statistically reliable results.

## 3 Results

### 3.1 Bidirectional causality between vegetarianism and depressive symptoms

**Figure 2:**
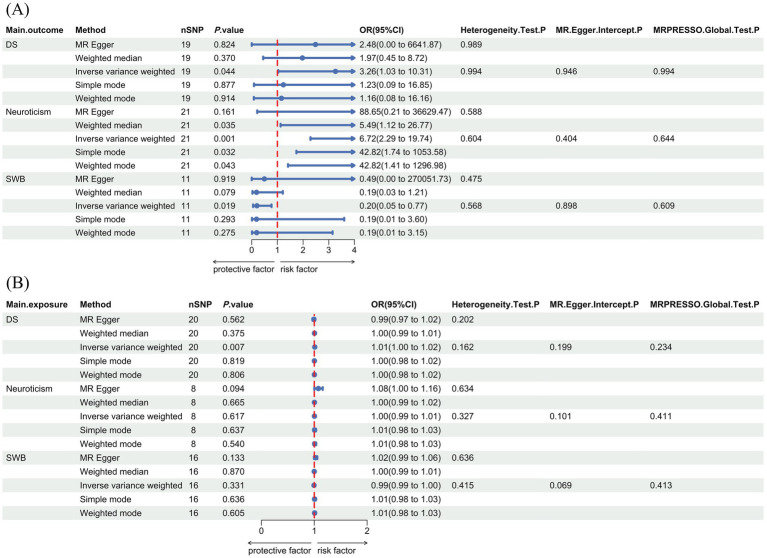
Forest plot with main MR results and sensitivity analysis results. (A) The forward MR analyses results (using vegetarianism as exposure). (B) The reverse MR analyses results (using vegetarianism as the outcome). DS, depressive symptoms; SWB: subjective well-being; OR, odds ratio; CI, confidence interval; SNPs, single nucleotide polymorphisms.

After a rigorous selection process, 19 SNPs were used as IVs when vegetarianism was the exposure, and 20 SNPs were selected as IVs when depressive symptoms were treated as exposure. The F statistics of all SNPs exceeded 10. Detailed information on IVs is provided in Supplementary Table S1. Using the IVW method, results indicated that vegetarianism was a potential risk factor for depressive symptoms (IVW: OR, 3.26; 95% CI, 1.03–10.31; *p* = 0.044). The reverse MR results indicated that depressive symptoms play a potential causal role in vegetarianism (IVW: OR, 1.01; 95% CI, 1.00–1.02; *p* = 6.87 × 10^−3^). The main MR results and forest plots are presented in Figure 2. Supplementary Figure S1 demonstrates the scatter plots of MR analyses.

### 3.2 Causality between vegetarianism and neuroticism

When vegetarianism was the exposure, 21 SNPs were selected as IVs for forward MR analysis. When neuroticism was the exposure, eight SNPs were selected as IVs for reverse MR analysis. The F statistics of all selected SNPs were greater than 10. Detailed information on each SNP used as IVs is provided in Supplementary Table S1. MR analyses suggested a positive causal relationship between vegetarianism and neuroticism (IVW: OR, 6.72; 95% CI, 2.29–19.74; *p* = 5.31 × 10^−4^), while there was no evidence to suggest that neuroticism played a causal role in vegetarianism (IVW: OR, 1.00; 95% CI, 0.99–1.01; *p* = 0.617). The MR results and forest plots are presented in Figure 2. Supplementary Figure S1 shows the scatter plots of MR analyses.

### 3.3 Causality between vegetarianism and subjective well-being

After selection, 11 SNPs were used as IVs in the forward MR analyses with vegetarianism as the exposure, and 16 SNPs were used as IVs in the reverse MR analyses with SWB as the exposure. The F statistics of all selected SNPs were greater than 10. Detailed information on these IVs is provided in Supplementary Table S1. MR analysis results indicated that vegetarianism is a potential risk factor for reducing SWB (IVW: OR, 0.20; 95% CI, 0.05–0.77; *p* = 0.019), while there was no evidence to suggest that SWB played a causal role in vegetarianism (IVW: OR, 0.99; 95% CI, 0.99–1.00; *p* = 0.331). The MR results and forest plots are presented in Figure 2. Supplementary Figure S1 shows the scatter plots of MR analyses.

### 3.4 Sensitivity analyses

None of the six MR analyses showed significant heterogeneity or pleiotropy. Figure 2 illustrates the results of the main heterogeneity and pleiotropy tests of six MR analyses. The Cochran’s Q test suggested no significant heterogeneity (*p* > 0.05). Both the MR-Egger pleiotropy and MR-PRESSO global tests did not reveal significant pleiotropy (*p* > 0.05). The results of the “leave-one-out” analysis are shown in Supplementary Figure S2.

### 3.5 Bias and type I error of sample overlap

The sample overlap rates for SWB, neuroticism, and depressive symptoms were 9.16%, 24.23%, and 23.89%, respectively. Given that the overlap rates for depressive symptoms and neuroticism exceeded 10%, we conducted bias estimations. For depressive symptoms, the estimated bias was 0.034, with the type I error rate of 0.05. For neuroticism, the estimated bias was 0.029, with the type I error rate of 0.06.

### 3.6 Statistical power

The statistical powers of all three forward MR analyses were 100%. Based on previous studies, the statistical power of forward MR analyses indicated a high possibility of discovering significant results in our study (41).

## 4 Discussion

This study is the first to employ bidirectional two-sample MR analyses to investigate the causal relationships between genetically predicted vegetarianism and depressive symptoms, neuroticism, and SWB. The results suggested that vegetarianism is a potential risk factor for depressive symptoms, neuroticism, and lower SWB. Besides, results also indicated a bidirectional causal relationship between vegetarianism and depressive symptoms.

Our findings revealed a bidirectional relationship between vegetarianism and depressive symptoms, indicating the complexity of these interactions. Several observational studies also reported similar results. Recently, a study of Peruvian adults reported vegetarians had more depressive symptoms than non-vegetarians (42). An investigation involving 9,668 adult male partners of pregnant women found that vegetarians had, on average, higher depression scores than non-vegetarians (43). Matta et al. (44) reported that depressive symptoms were associated with the exclusion of any food group, including meat. However, conflicting evidence also exists regarding the associations between vegetarianism and depressive symptoms. Askari et al. (45) analyzed the pooled effect values from 10 cohort studies and found no significant associations between vegetarianism and depression. An investigation among South Asians in the United States reported that the odds of depression were 43% lower among vegetarians (46). A similar conclusion was drawn in a prospective cohort study of a Taiwanese population (47). Their conclusions were opposite to ours, possibly because the sample sizes they used were smaller, and the traits of the sample population were different.

Our study results indicated that vegetarianism plays a potential role in neuroticism, which was also supported by previous research findings. A study in 2018 reported that vegetarians were more likely to be neurotic and depressed (48). Additionally, a study in 2023 suggested that individuals with neurotic and agreeable personalities had a lower frequency of poultry consumption (49). However, in a hierarchical regression analysis conducted in an Australian population, consuming plant-based food was associated with greater emotional stability (50). Different results might be related to the variations in study design and the phenotypic characteristics.

A survey on the lifestyle and mental health of Chinese and German students found that a vegetarian diet was associated with lower positive mental health (15), similar to the results observed in a study of 9,113 Australian women (14). Since poor mental health typically correlates with lower SWB, these findings align with our conclusions. However, a cross-sectional study of 138 Seventh-Day Adventist adults found that vegetarians experienced better moods compared to omnivores (16). This discrepancy might be attributed to the unique lifestyle habits of the sample population. Besides, another study suggested that a worksite vegan nutrition program could improve physical health, mental health, and overall diet satisfaction (17). This program was guided by professionals, and participants were advised to take vitamin B_12_ supplements, which may explain the differences in results compared to our study.

A plant-based diet may lack essential nutrients such as vitamin B_12_, vitamin D, calcium, and long-chain *ω*-3 polyunsaturated fatty acid (PUFA), which can negatively impact the health of those following an unbalanced vegetarian diet (51). These nutrients play critical roles in brain and nervous system function (52). For example, vitamin B_12_ is almost absent in plant-based food, so its deficiency is common among vegetarians (53). Low vitamin B_12_ level is associated with the risk of depression, as supported by multiple studies (54–56). Vitamin B_12_ deficiency may result in several neuropsychiatric conditions, including Alzheimer’s disease, dementia, weakness, memory loss, irritability, and personality changes, all of which can significantly reduce SWB (57, 58). Additionally, a deficiency in *ω*-3 PUFAs is also a potential mechanism of the vegetarians’ negative mental well-being. Eicosapentaenoic acid (EPA) and docosahexaenoic acid (DHA), the main members of ω-3 PUFA families, mainly come from fish and fish oil (59). One study has discovered that adding E-EPA to antidepressant therapy can significantly improve depressive symptoms in patients with unipolar depressive disorder (60), and a recent randomized controlled clinical trial involving 71 adolescents with depression also reported similar findings (61). Dietary intake of ω-3 PUFAs is negatively correlated with the risk of depression (62). ω-3 PUFAs exert their antidepressant effects potentially through anti-inflammatory functions and by influencing the quantity and biological effects of neurotransmitters (59). In contemporary diets, the high prevalence of fortified foods can help vegetarians replenish their deficient nutrients promptly, thereby protecting their mental health. Thus, social psychological factors may also play a crucial role.

Social pressure and cultural conflicts play a significant role in poor mental well-being associated with vegetarianism. As a minority dietary choice in a predominantly carnivorous culture, vegetarianism can cause various negative impacts on the social lives of vegetarians, including bias and discrimination from omnivores, lack of understanding from friends and family, anxiety and stress due to difficulties in making food choices at work or social gatherings, conflicts of values and ethics with the dominant culture, and more (63). Such experiences can lead to feelings of loneliness, anxiety, self-doubt, and other negative feelings, that contribute to depressive symptoms, neuroticism, and lower SWB. Besides, the motivations behind choosing vegetarianism are also related to vegetarians’ mental well-being. In Western countries, concerns about the environment, animals, and personal health are the most common motivations (64). Studies found that omnivores tend to show defensive stereotypes and bias toward those who choose a vegetarian or vegan diet for ethical reasons, which can clearly harm vegetarians’ mental health (65–67). Additionally, economic factors can influence vegetarians’ mental health differently depending on income levels. Individuals who are forced into vegetarianism due to economic hardship may derive little satisfaction from their diet; instead, their mental health may suffer due to inadequate nutrition (68). While those with higher incomes can afford better-quality produce for their health and ethic motivations. However, even high-income vegetarians may still face the pressure of stereotypes, as in some cultures, meat consumption symbolizes wealth and status (69).

Focusing on the contradictions in previous studies, this study employed the MR approach to offer valuable insights into the future psychological health management of vegetarians. It is essential to monitor the mental health of vegetarians and consider preventive measures to mitigate depressive symptoms and other adverse effects. Timely supplementation of nutrients commonly deficient in vegetarian diets serves as an effective strategy. Furthermore, our findings provide some ideas for strengthening dietary management to improve mental well-being.

Our study has several strengths. First, the GWAS data used had large sample sizes, and the GWAS of depressive symptoms, neuroticism, and SWB were sourced from large consortia. Second, all samples were drawn from European populations, which reduces bias due to population stratification. Third, the analysis revealed no significant heterogeneity or pleiotropy. Fourth, the SNPs used as IVs underwent *F*-value calculations, ensuring that weak instruments were excluded (*F* > 10). Finally, our study used genetically predicted vegetarianism as the exposure and analyzed three psychological states (depressive symptoms, neuroticism, and SWB) as outcomes. We also conducted reverse MR analysis, providing broader evidence of the association between vegetarianism and mental health from multiple perspectives.

This study also has several limitations. First, the data were sourced exclusively from European populations, which may limit the generalizability of our findings to other populations. Second, a small portion of the exposure and outcome data came from the UKB, leading to potential sample overlap that could introduce bias. Consequently, we ensured that all SNPs used were strong IVs and estimated the bias and type I error rates for phenotypes with sample overlaps exceeding 10%. Third, the genetic associations were based on self-reported data, potentially introducing recall and subjective biases. Fourth, MR analyses are limited to inferring linear causal relationships and cannot evaluate time-varying causality, as genetic variants have cumulative, lifelong effects and studies may not fully capture the time-varying nature of the exposures. Additionally, vegetarianism includes varying degrees of dietary restrictions on animal products, resulting in multiple classifications. There may also be inconsistencies between how people self-identify and the definitions found in the literature (64). Future studies should expand data sources and conduct a more detailed exploration. Lastly, the influence of related biological and social factors on the observed associations requires further exploration.

## 5 Conclusion

MR analyses indicate causal associations between genetically predicted vegetarianism and mental well-being, shown by an increased risk of depressive symptoms and neuroticism, along with a decrease in SWB. Additionally, MR analysis suggests a potential bidirectional causal relationship between vegetarianism and depressive symptoms. These conclusions are based on data from European populations. The findings suggest that mental health is an important aspect to consider in the lifestyle and dietary choices of vegetarians.

## Data Availability

The original contributions presented in the study are included in the article/Supplementary material, further inquiries can be directed to the corresponding author.

## Footnotes

^1^ https://www.R-project.org

^2^ https://sb452.shinyapps.io/overlap/

^3^ https://shiny.cnsgenomics.com/mRnd/
