# Supplementary material for: Using genetic variations to reveal the complex relationships between vegetarianism and well-being, depressive symptoms and neuroticism
Source: Front Nutr. 2024 Oct 15;11:1389000. doi: 10.3389/fnut.2024.1389000 (PMC11518772; doi:10.3389/fnut.2024.1389000)
Supplement: Supplementary file 1 [file Data_Sheet_1.docx]

Supplementary Material

# Supplementary Figures

## **Supplementary Figure S1.** Scatter plots of Mendelian Randomization (MR) analyses. Black blots show the individual inverse variance (IV) associations with exposure and outcome. The vertical and horizontal lines show the 95% CI of the odds ratio. The slope of the lines shows the estimated causal effect of the MR methods.

### Scatter plot of MR analysis f**rom vegetarianism** to depressive symptoms.


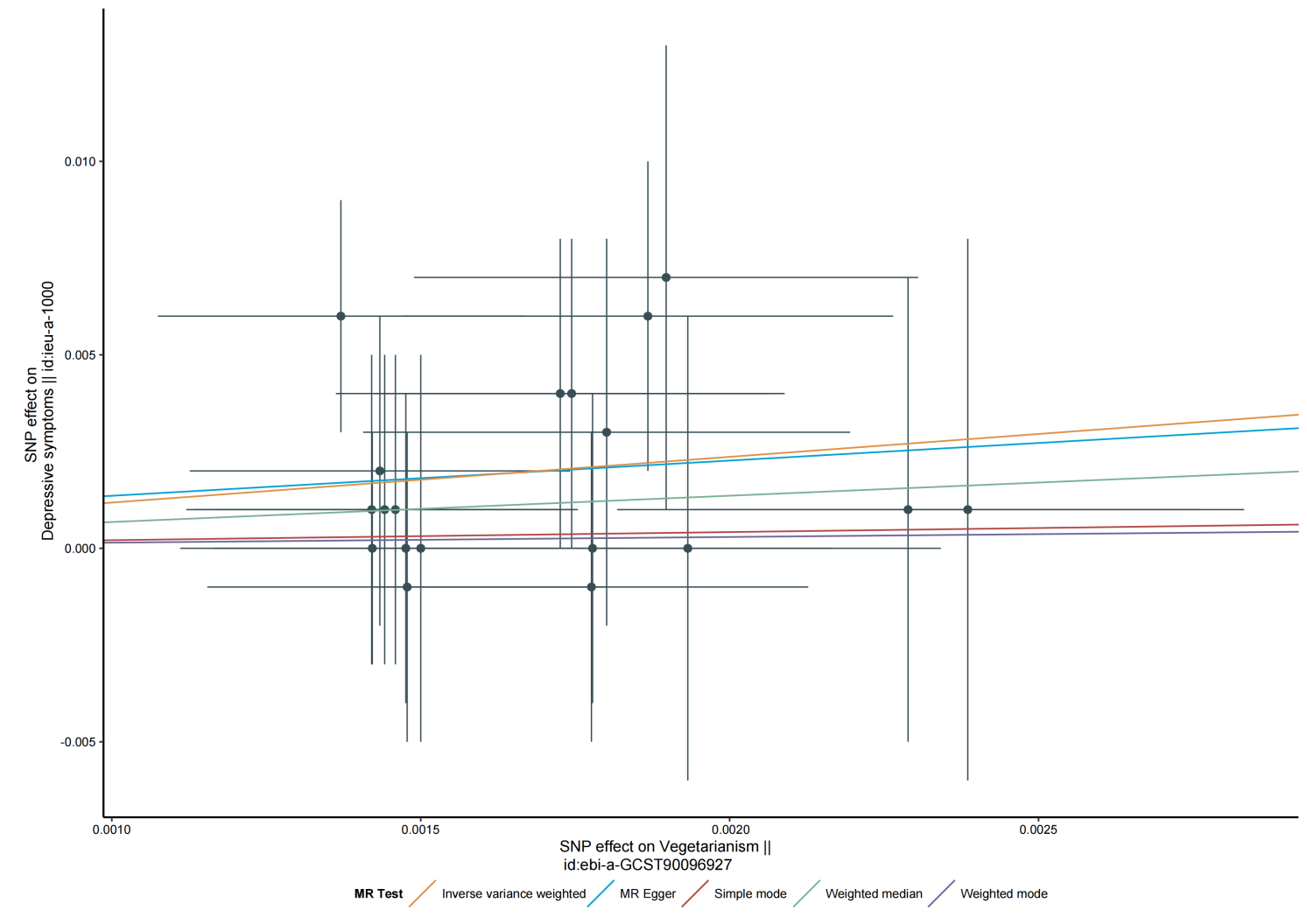


### Scatter plot of MR analysis **from** depressive symptoms to vegetarianism.


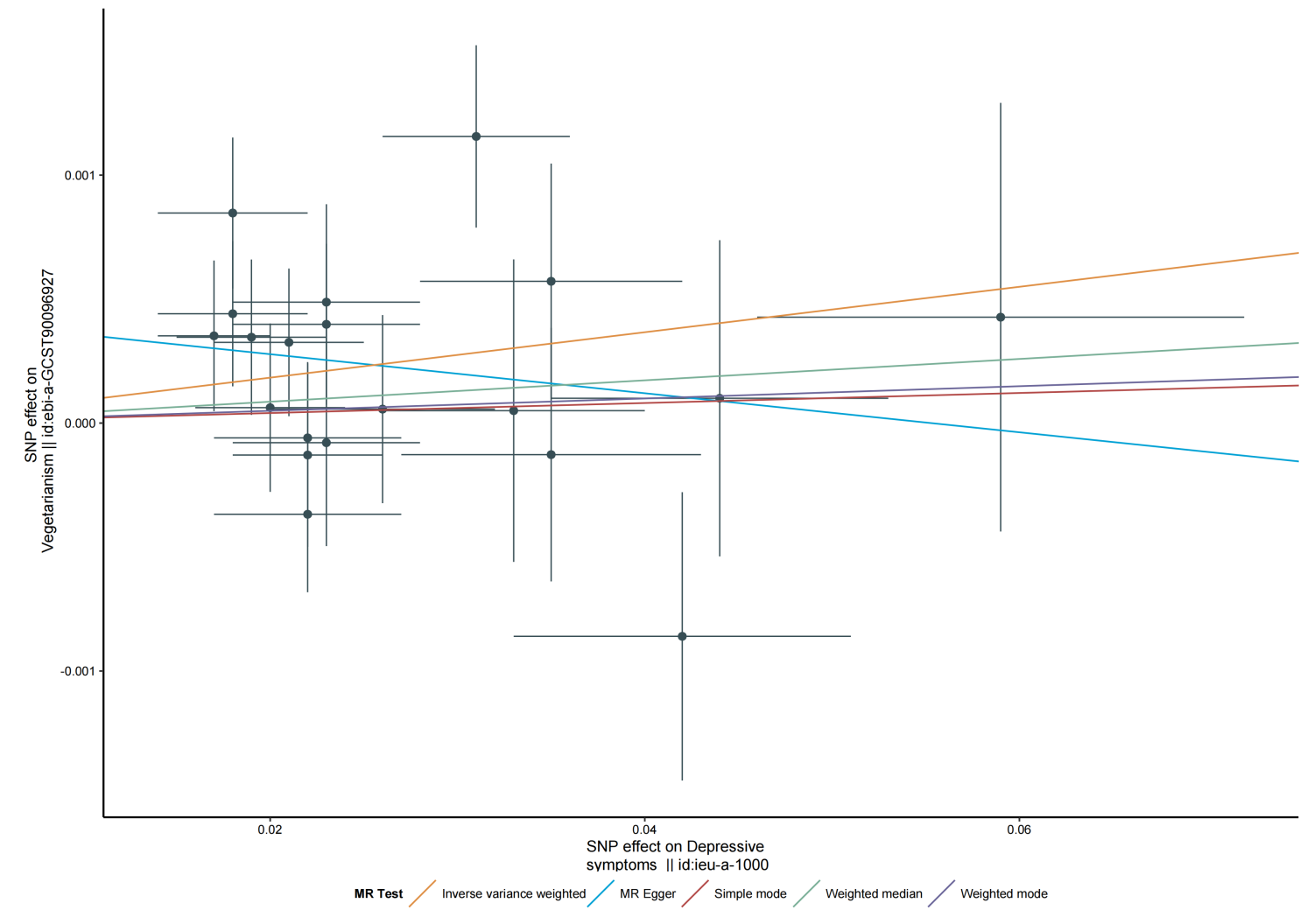


### Scatter plot of MR analysis from vegetarianism to neuroticism.

**
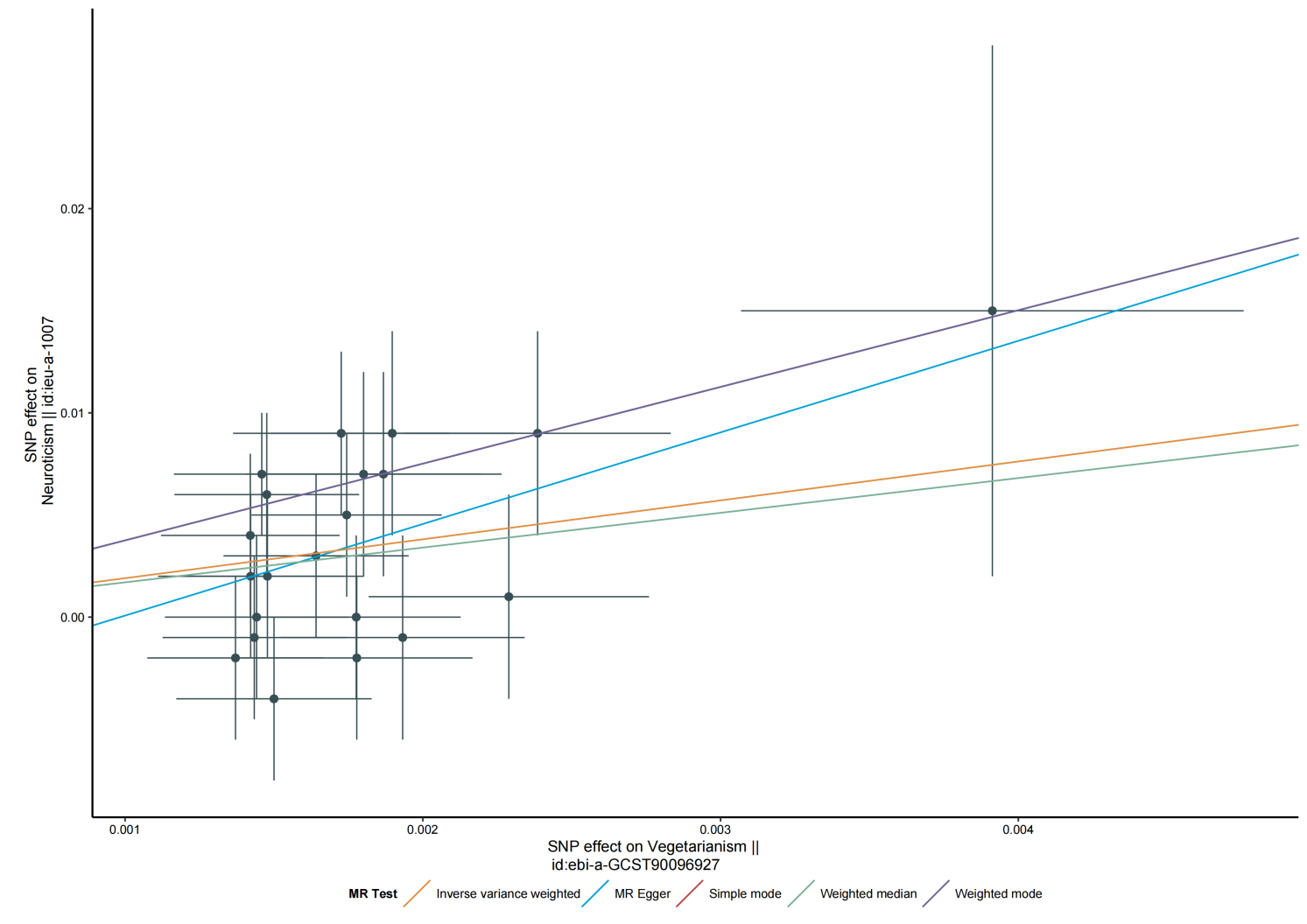
**

### Scatter plot of MR analysis **from** neuroticism to vegetarianism.

**
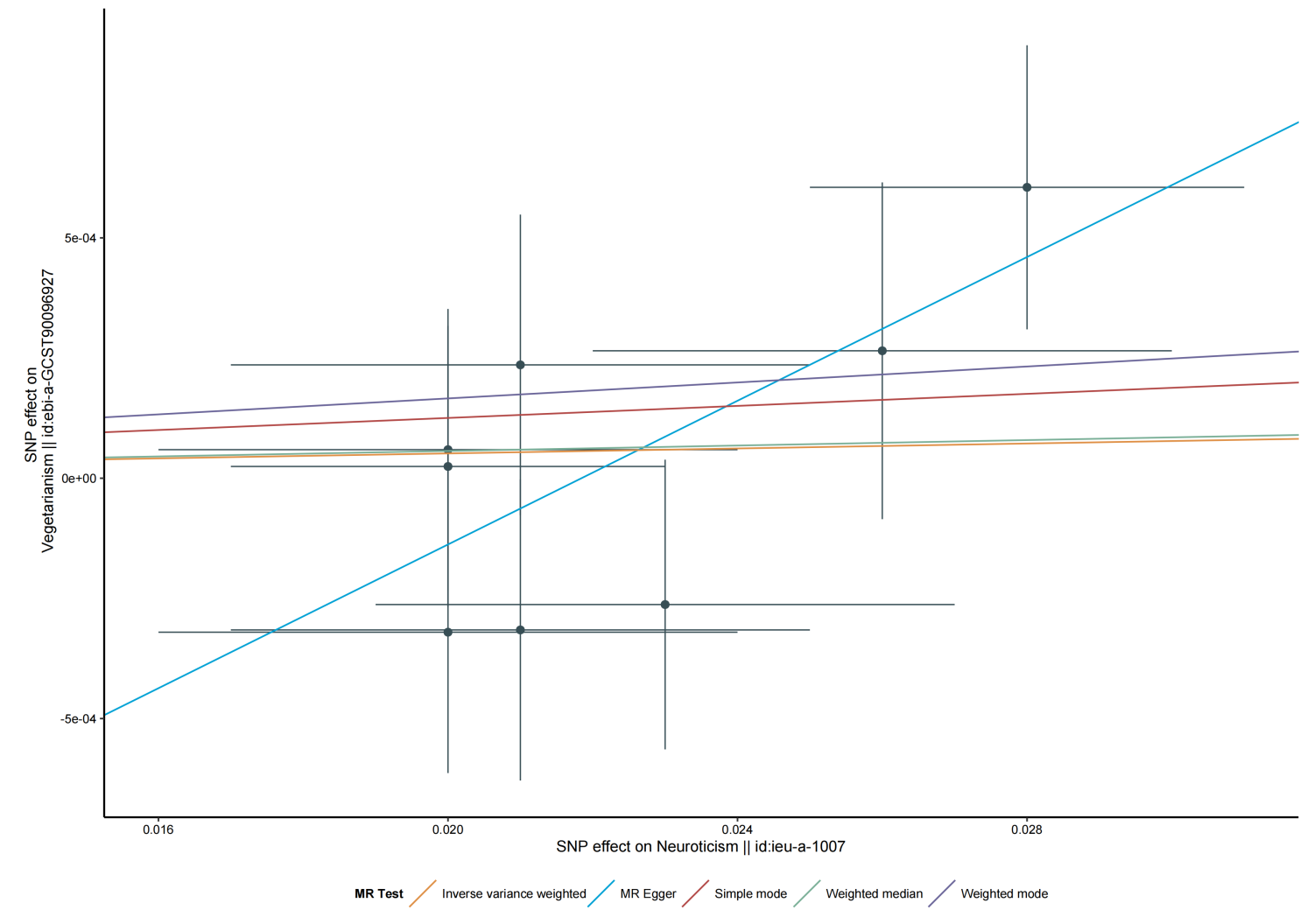
**

### Scatter plot of MR analysis from vegetarianism to subjective well-being.


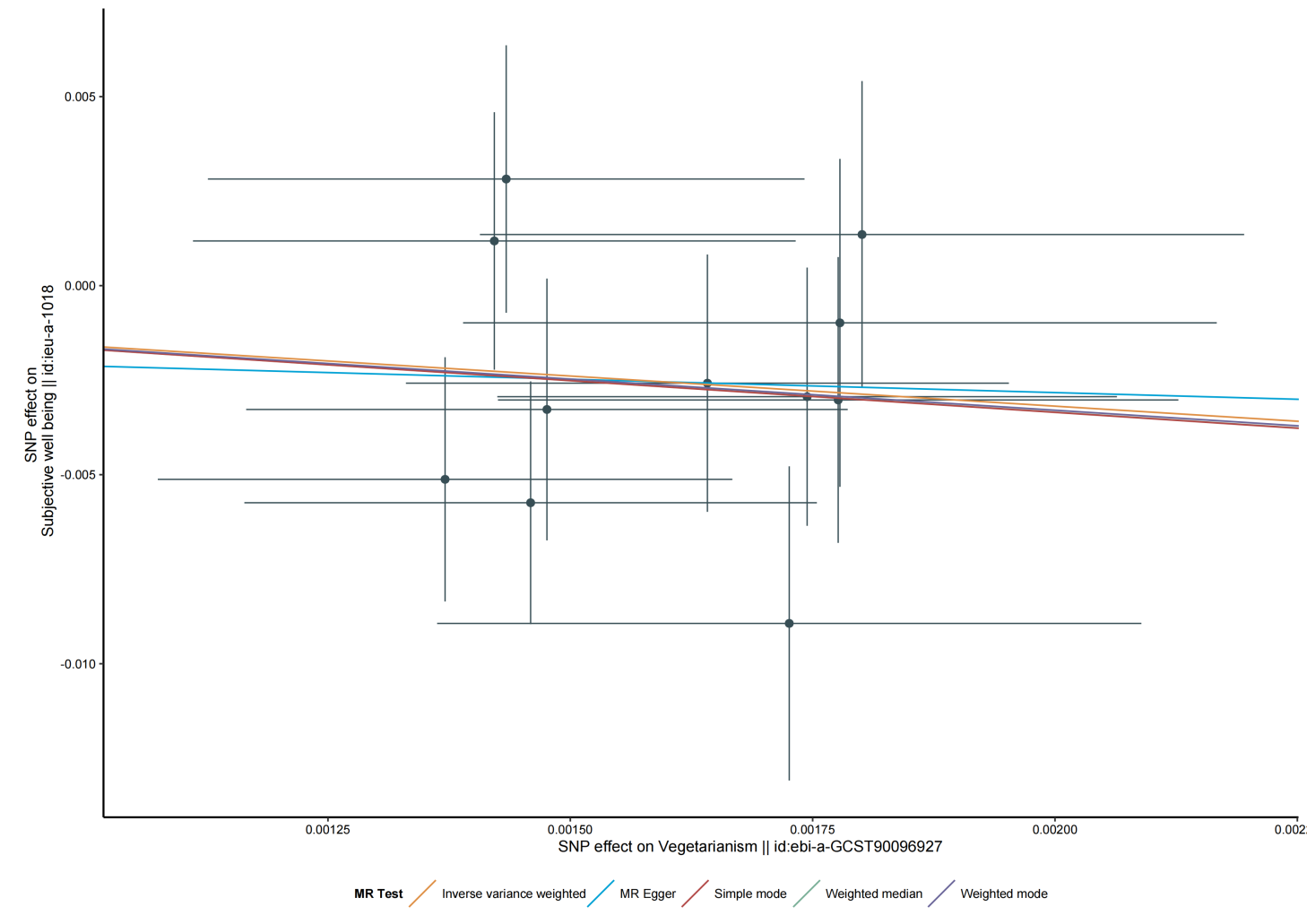


### Scatter plot of MR analysis from subjective well-being to vegetarianism.

**
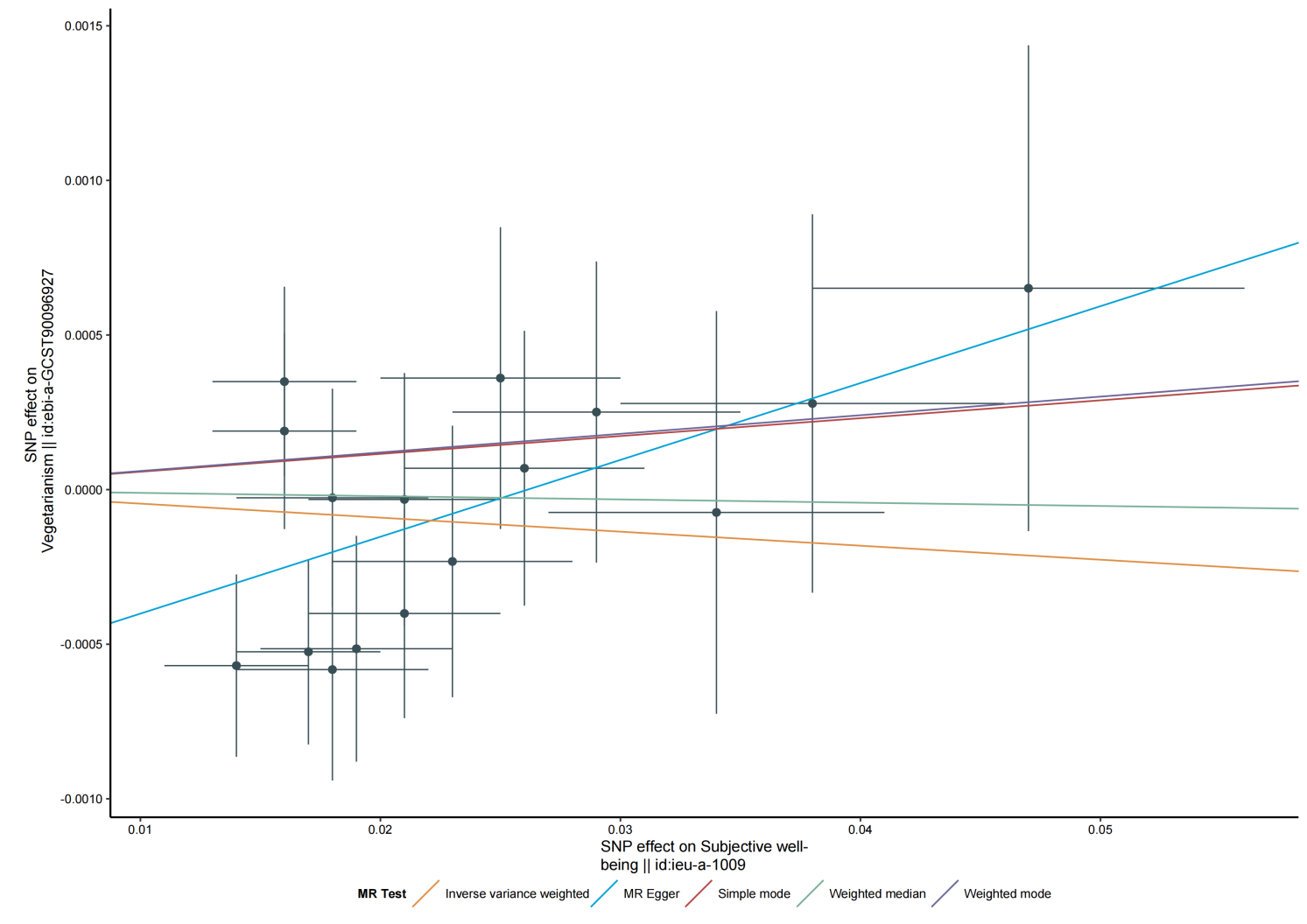
**

## **Supplementary Figure S2. Leave-one-out sensitivity analyses of Mendelian randomization (MR) analyses.** Each black spots shows the inverse variance weighted (IVW) result of analysis after the particular Single Nucleotide Polymorphism (SNP) was excluded. The red spot shows the IVW result using all SNPs.

### **Leave-one-out sensitivity analysis of MR analysis from vegetarianism** to depressive symptoms.


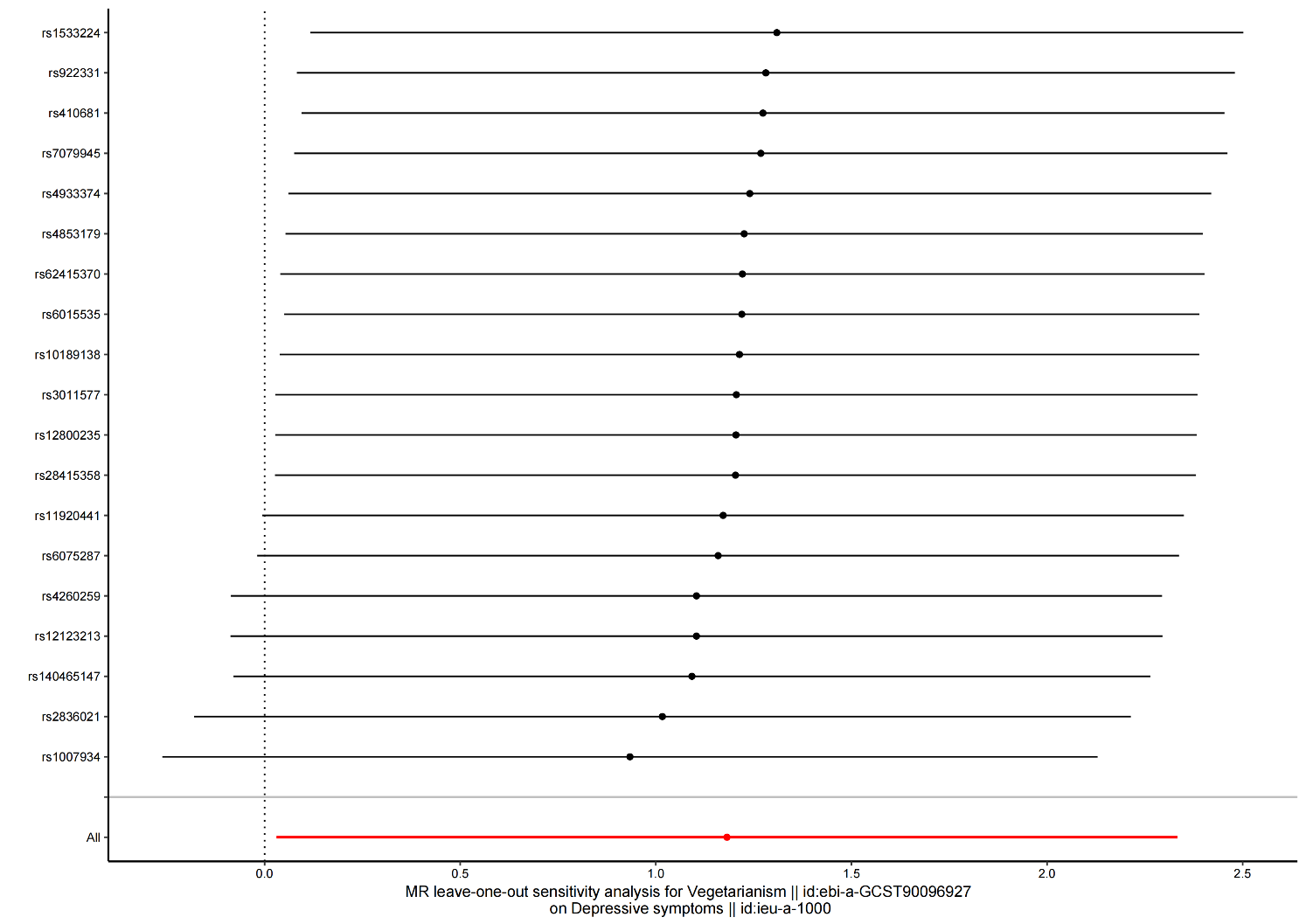


### Leave-one-out sensitivity analysis of MR analysis from depressive symptoms to vegetarianism.


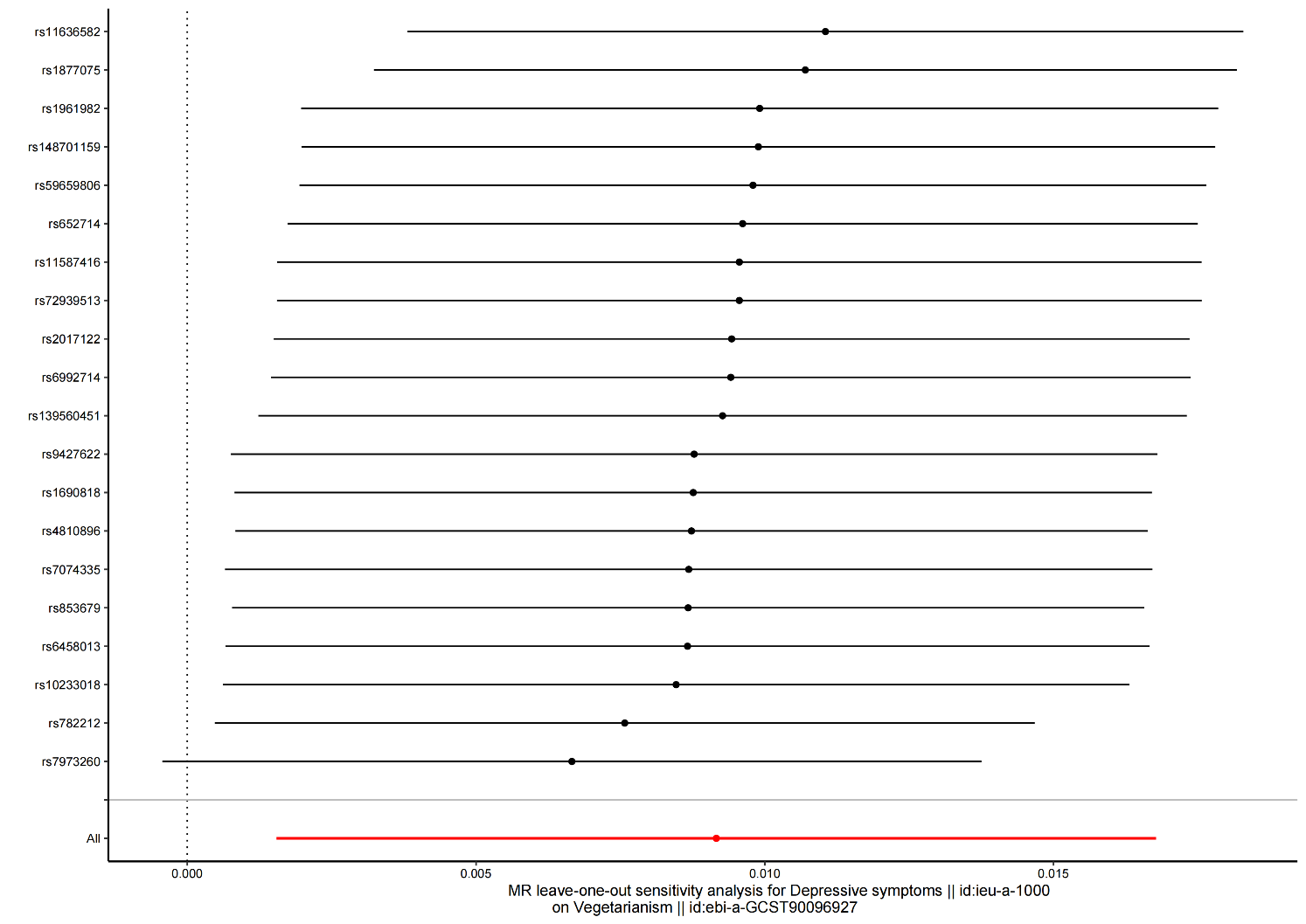


### Leave-one-out sensitivity analysis of MR analysis from vegetarianism to neuroticism.


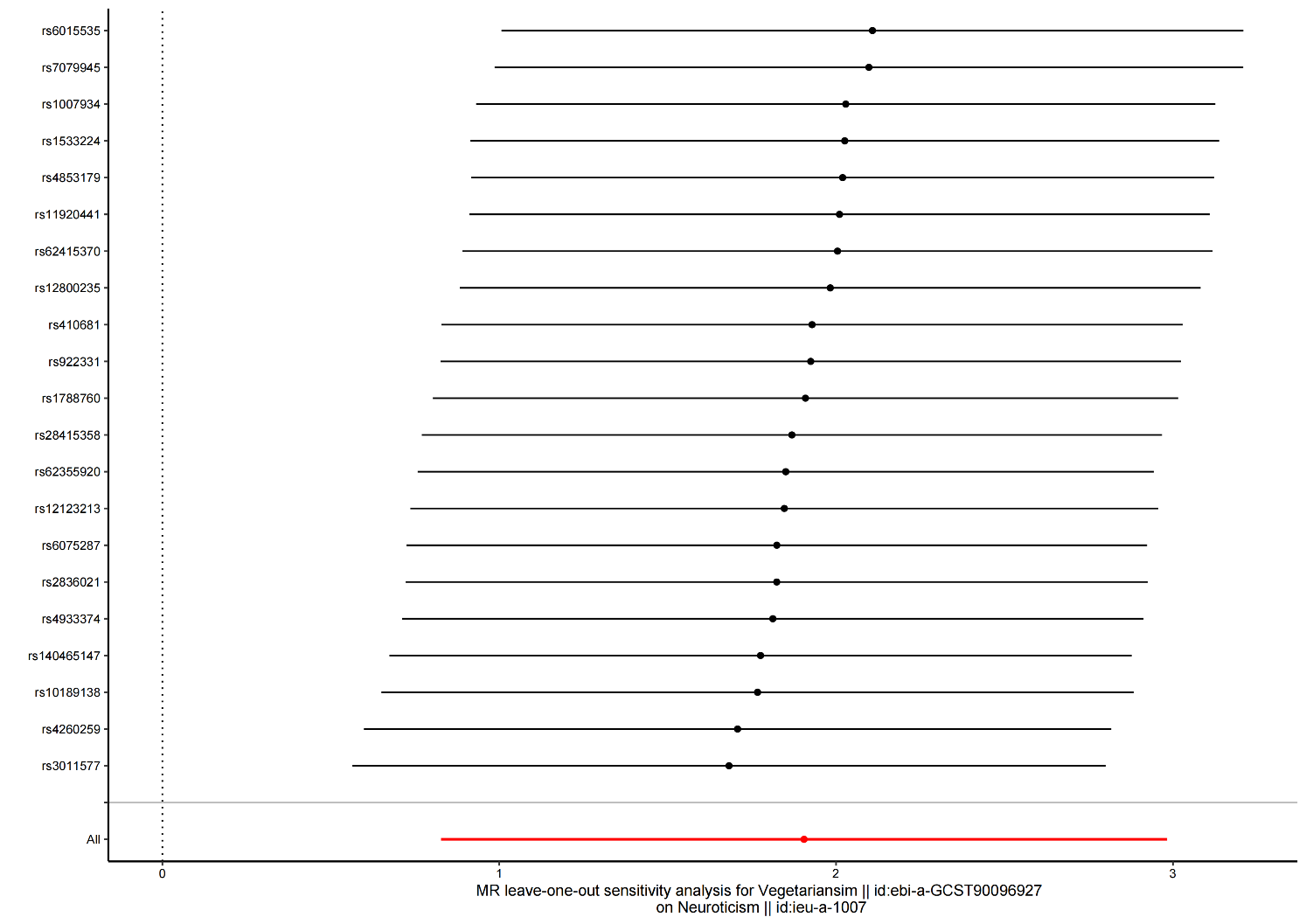


### Leave-one-out sensitivity analysis of MR analysis from neuroticism to vegetarianism.


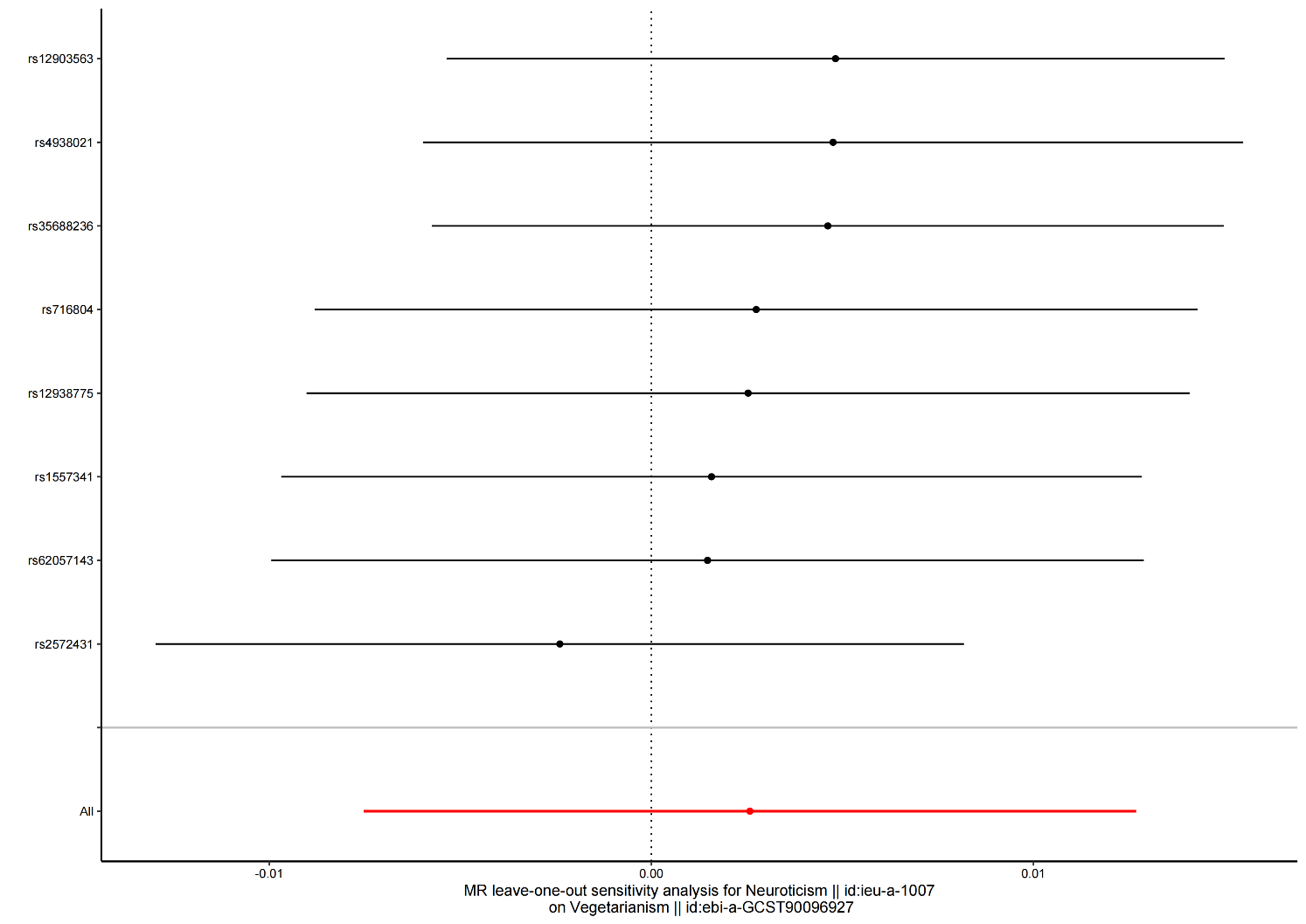


### Leave-one-out sensitivity analysis of MR analysis from vegetarianism to subjective well-being.


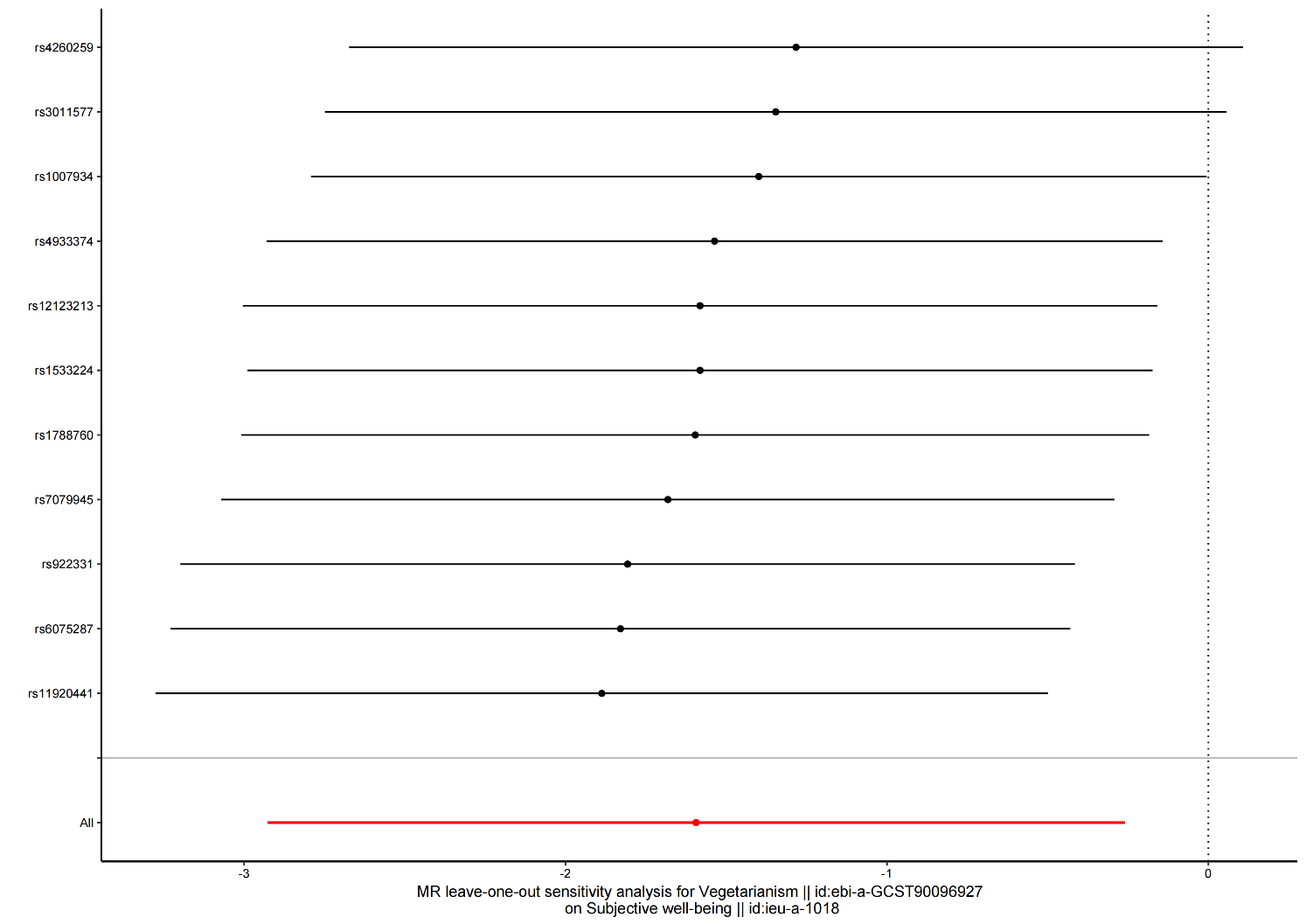


### **Leave-one-out sensitivity analysis of MR analysis from subjective well-**being to vegetarianism.


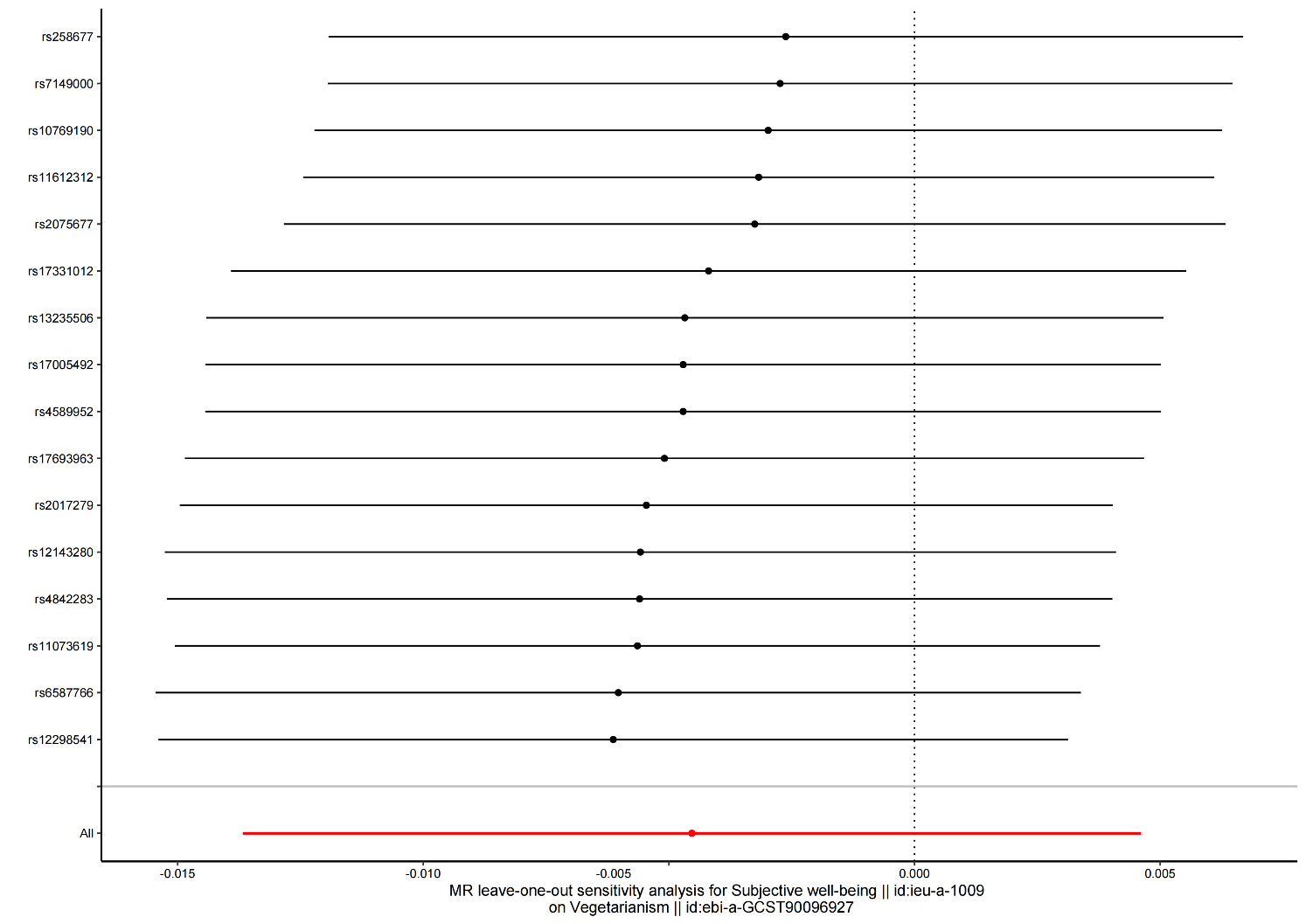


# Supplementary Tables

## Supplementary Table S1. Final Single Nucleotide Polymorphisms (SNPs) in each trait used in Mendelian randomization(MR) analysis.

### vegetarianism || ebi-a-GCST90096927 on depressive symptoms || ieu-a-1000 (19-SNP-P< 5.0x10^-6^)

| SNPID | chr | pos | effect_allele | other_allele | beta.exposure | se.exposure | *p*.exposure | beta.outcome | se.outcome | *p*.outcome | F |
| --- | --- | --- | --- | --- | --- | --- | --- | --- | --- | --- | --- |
| rs1007934 | 14 | 73463479 | A | G | 1.37E-03 | 2.96E-04 | 3.73E-06 | 0.006 | 0.003 | 0.083 | 21.397 |
| rs10189138 | 2 | 58153676 | T | G | 2.39E-03 | 4.47E-04 | 9.70E-08 | 0.001 | 0.007 | 0.864 | 28.433 |
| rs11920441 | 3 | 51100020 | C | T | -1.43E-03 | 3.08E-04 | 3.17E-06 | -0.002 | 0.004 | 0.655 | 21.709 |
| rs12123213 | 1 | 74165735 | G | A | -1.74E-03 | 3.20E-04 | 4.83E-08 | -0.004 | 0.004 | 0.275 | 29.784 |
| rs12800235 | 11 | 43747912 | G | A | -1.44E-03 | 3.08E-04 | 2.87E-06 | -0.001 | 0.004 | 0.784 | 21.904 |
| rs140465147 | 8 | 1137979 | C | T | -1.90E-03 | 4.08E-04 | 3.31E-06 | -0.007 | 0.006 | 0.252 | 21.63 |
| rs1533224 | 11 | 80584598 | A | C | -1.78E-03 | 3.51E-04 | 4.17E-07 | 0.001 | 0.004 | 0.832 | 25.616 |
| rs2836021 | 21 | 39217408 | C | T | 1.87E-03 | 3.97E-04 | 2.54E-06 | 0.006 | 0.004 | 0.170 | 22.135 |
| rs28415358 | 7 | 24504481 | G | A | 1.42E-03 | 3.00E-04 | 2.18E-06 | 0.001 | 0.004 | 0.797 | 22.425 |
| rs3011577 | 1 | 238949005 | T | C | -1.46E-03 | 2.95E-04 | 7.78E-07 | -0.001 | 0.004 | 0.814 | 24.411 |
| rs410681 | 6 | 42670469 | A | G | -1.48E-03 | 3.24E-04 | 4.92E-06 | 0.001 | 0.004 | 0.888 | 20.869 |
| rs4260259 | 2 | 165133462 | A | G | -1.73E-03 | 3.63E-04 | 2.03E-06 | -0.004 | 0.004 | 0.383 | 22.571 |
| rs4853179 | 2 | 70175240 | T | G | -1.93E-03 | 4.10E-04 | 2.40E-06 | 0 | 0.006 | 0.971 | 22.244 |
| rs4933374 | 10 | 87317945 | G | A | -1.48E-03 | 3.10E-04 | 1.97E-06 | 0 | 0.004 | 0.924 | 22.627 |
| rs6015535 | 20 | 58203911 | T | C | -1.50E-03 | 3.28E-04 | 4.82E-06 | 0 | 0.005 | 0.999 | 20.907 |
| rs6075287 | 20 | 18103057 | A | G | -1.80E-03 | 3.94E-04 | 4.91E-06 | -0.003 | 0.005 | 0.529 | 20.872 |
| rs62415370 | 6 | 67152120 | A | C | -2.29E-03 | 4.71E-04 | 1.17E-06 | -0.001 | 0.006 | 0.827 | 23.629 |
| rs7079945 | 10 | 129247444 | T | G | -1.78E-03 | 3.89E-04 | 4.79E-06 | 0 | 0.004 | 0.917 | 20.92 |
| rs922331 | 3 | 192848225 | C | T | 1.42E-03 | 3.11E-04 | 4.81E-06 | 0 | 0.003 | 0.916 | 20.913 |

### vegetarianism || ebi-a-GCST90096927 on neuroticism || ieu-a-1007 (21-SNP-P< 5.0x10^-6^)

| SNPID | chr | pos | effect_allele | other_allele | beta.exposure | se.exposure | *p*.exposure | beta.outcome | se.outcome | *p*.outcome | F |
| --- | --- | --- | --- | --- | --- | --- | --- | --- | --- | --- | --- |
| rs1007934 | 14 | 73463479 | A | G | 1.37E-03 | 2.96E-04 | 3.73E-06 | -0.002 | 0.004 | 0.598 | 21.397 |
| rs10189138 | 2 | 58153676 | T | G | 2.39E-03 | 4.47E-04 | 9.70E-08 | 0.009 | 0.005 | 0.061 | 28.433 |
| rs11920441 | 3 | 51100020 | C | T | -1.43E-03 | 3.08E-04 | 3.17E-06 | 0.001 | 0.004 | 0.795 | 21.709 |
| rs12123213 | 1 | 74165735 | G | A | -1.74E-03 | 3.20E-04 | 4.83E-08 | -0.005 | 0.004 | 0.219 | 29.784 |
| rs12800235 | 11 | 43747912 | G | A | -1.44E-03 | 3.08E-04 | 2.87E-06 | 0 | 0.004 | 0.970 | 21.904 |
| rs140465147 | 8 | 1137979 | C | T | -1.90E-03 | 4.08E-04 | 3.31E-06 | -0.009 | 0.005 | 0.080 | 21.630 |
| rs1533224 | 11 | 80584598 | A | C | -1.78E-03 | 3.51E-04 | 4.17E-07 | 0 | 0.004 | 0.940 | 25.616 |
| rs1788760 | 18 | 21137442 | G | A | 1.64E-03 | 3.11E-04 | 1.31E-07 | 0.003 | 0.004 | 0.425 | 27.853 |
| rs2836021 | 21 | 39217408 | C | T | 1.87E-03 | 3.97E-04 | 2.54E-06 | 0.007 | 0.005 | 0.145 | 22.135 |
| rs28415358 | 7 | 24504481 | G | A | 1.42E-03 | 3.00E-04 | 2.18E-06 | 0.004 | 0.004 | 0.222 | 22.425 |
| rs3011577 | 1 | 238949005 | T | C | -1.46E-03 | 2.95E-04 | 7.78E-07 | -0.007 | 0.003 | 0.059 | 24.411 |
| rs410681 | 6 | 42670469 | A | G | -1.48E-03 | 3.24E-04 | 4.92E-06 | -0.002 | 0.004 | 0.515 | 20.869 |
| rs4260259 | 2 | 165133462 | A | G | -1.73E-03 | 3.63E-04 | 2.03E-06 | -0.009 | 0.004 | 0.038 | 22.571 |
| rs4853179 | 2 | 70175240 | T | G | -1.93E-03 | 4.10E-04 | 2.40E-06 | 0.001 | 0.005 | 0.772 | 22.244 |
| rs4933374 | 10 | 87317945 | G | A | -1.48E-03 | 3.10E-04 | 1.97E-06 | -0.006 | 0.004 | 0.081 | 22.627 |
| rs6015535 | 20 | 58203911 | T | C | -1.50E-03 | 3.28E-04 | 4.82E-06 | 0.004 | 0.004 | 0.274 | 20.907 |
| rs6075287 | 20 | 18103057 | A | G | -1.80E-03 | 3.94E-04 | 4.91E-06 | -0.007 | 0.005 | 0.117 | 20.872 |
| rs62355920 | 5 | 107104175 | C | T | 3.91E-03 | 8.44E-04 | 3.55E-06 | 0.015 | 0.013 | 0.246 | 21.493 |
| rs62415370 | 6 | 67152120 | A | C | -2.29E-03 | 4.71E-04 | 1.17E-06 | -0.001 | 0.005 | 0.888 | 23.629 |
| rs7079945 | 10 | 129247444 | T | G | -1.78E-03 | 3.89E-04 | 4.79E-06 | 0.002 | 0.004 | 0.600 | 20.920 |
| rs922331 | 3 | 192848225 | C | T | 1.42E-03 | 3.11E-04 | 4.81E-06 | 0.002 | 0.004 | 0.530 | 20.913 |

### vegetarianism || ebi-a-GCST90096927 on subjective well-being || ieu-a-1009 (11-SNP-P< 5.0x10^-6^)

| SNPID | chr | pos | effect_allele. | other_allele | beta.exposure | se.exposure | *p*.exposure | beta.outcome | se.outcome | *p*.outcome | F |
| --- | --- | --- | --- | --- | --- | --- | --- | --- | --- | --- | --- |
| rs1007934 | 14 | 73463479 | A | G | 1.37E-03 | 2.96E-04 | 3.73E-06 | -5.12E-03 | 3.23E-03 | 0.113 | 21.397 |
| rs11920441 | 3 | 51100020 | C | T | -1.43E-03 | 3.08E-04 | 3.17E-06 | -2.82E-03 | 3.54E-03 | 0.425 | 21.709 |
| rs12123213 | 1 | 74165735 | G | A | -1.74E-03 | 3.20E-04 | 4.83E-08 | 2.94E-03 | 3.42E-03 | 0.39 | 29.784 |
| rs1533224 | 11 | 80584598 | A | C | -1.78E-03 | 3.51E-04 | 4.17E-07 | 3.02E-03 | 3.78E-03 | 0.424 | 25.616 |
| rs1788760 | 18 | 21137442 | G | A | 1.64E-03 | 3.11E-04 | 1.31E-07 | -2.58E-03 | 3.40E-03 | 0.449 | 27.853 |
| rs3011577 | 1 | 238949005 | T | C | -1.46E-03 | 2.95E-04 | 7.78E-07 | 5.74E-03 | 3.21E-03 | 0.074 | 24.411 |
| rs4260259 | 2 | 165133462 | A | G | -1.73E-03 | 3.63E-04 | 2.03E-06 | 8.93E-03 | 4.15E-03 | 0.032 | 22.571 |
| rs4933374 | 10 | 87317945 | G | A | -1.48E-03 | 3.10E-04 | 1.97E-06 | 3.28E-03 | 3.46E-03 | 0.344 | 22.627 |
| rs6075287 | 20 | 18103057 | A | G | -1.80E-03 | 3.94E-04 | 4.91E-06 | -1.35E-03 | 4.06E-03 | 0.739 | 20.872 |
| rs7079945 | 10 | 129247444 | T | G | -1.78E-03 | 3.89E-04 | 4.79E-06 | 9.85E-04 | 4.34E-03 | 0.821 | 20.92 |
| rs922331 | 3 | 192848225 | C | T | 1.42E-03 | 3.11E-04 | 4.81E-06 | 1.18E-03 | 3.41E-03 | 0.729 | 20.913 |

### Depressive symptoms || ieu-a-1000 on vegetarianism || ebi-a-GCST90096927 (20-SNP-P< 5.0x10^-6^)

| SNPID | chr | pos | effect_allele | other_allele | beta.exposure | se.exposure | *p*.exposure | beta.outcome | se.outcome | *p*.outcome | F |
| --- | --- | --- | --- | --- | --- | --- | --- | --- | --- | --- | --- |
| rs10233018 | 7 | 117523709 | G | A | 0.018 | 0.004 | 1.65E-07 | 4.41E-04 | 2.93E-04 | 0.132 | 20.250 |
| rs11587416 | 1 | 166509723 | T | C | -0.026 | 0.006 | 3.46E-06 | -5.63E-05 | 3.79E-04 | 0.882 | 18.778 |
| rs11636582 | 15 | 86599160 | T | G | -0.042 | 0.009 | 6.98E-07 | 8.60E-04 | 5.81E-04 | 0.139 | 21.778 |
| rs139560451 | 17 | 50446819 | T | C | -0.059 | 0.013 | 2.88E-06 | -4.27E-04 | 8.64E-04 | 0.621 | 20.598 |
| rs148701159 | 11 | 40148558 | G | A | -0.035 | 0.008 | 3.74E-06 | 1.27E-04 | 5.11E-04 | 0.803 | 19.141 |
| rs1690818 | 11 | 99496554 | T | C | -0.019 | 0.004 | 4.05E-07 | -3.46E-04 | 3.13E-04 | 0.269 | 22.563 |
| rs1877075 | 4 | 140925870 | A | G | -0.022 | 0.005 | 2.00E-06 | 3.68E-04 | 3.14E-04 | 0.242 | 19.360 |
| rs1961982 | 12 | 103612733 | A | G | -0.022 | 0.005 | 2.25E-06 | 5.97E-05 | 3.06E-04 | 0.845 | 19.360 |
| rs2017122 | 11 | 118314849 | T | C | 0.033 | 0.007 | 2.32E-06 | 5.01E-05 | 6.10E-04 | 0.935 | 22.224 |
| rs4810896 | 20 | 47535298 | C | A | 0.017 | 0.003 | 9.00E-07 | 3.52E-04 | 3.04E-04 | 0.247 | 32.111 |
| rs59659806 | 15 | 38919964 | C | T | 0.022 | 0.004 | 6.01E-07 | -1.29E-04 | 3.70E-04 | 0.728 | 30.250 |
| rs6458013 | 6 | 37687281 | T | C | 0.023 | 0.005 | 1.02E-06 | 3.98E-04 | 3.25E-04 | 0.220 | 21.160 |
| rs652714 | 18 | 53321026 | A | G | -0.023 | 0.005 | 8.95E-07 | 7.91E-05 | 4.17E-04 | 0.850 | 21.160 |
| rs6992714 | 8 | 64628120 | C | T | -0.02 | 0.004 | 9.32E-08 | -6.22E-05 | 3.40E-04 | 0.855 | 25.000 |
| rs7074335 | 10 | 106700394 | C | T | -0.035 | 0.007 | 4.57E-07 | -5.72E-04 | 4.75E-04 | 0.229 | 25.000 |
| rs72939513 | 1 | 83136096 | A | G | -0.044 | 0.009 | 3.14E-06 | -1.00E-04 | 6.38E-04 | 0.875 | 23.901 |
| rs782212 | 1 | 72945666 | T | C | -0.018 | 0.004 | 1.23E-06 | -8.47E-04 | 3.04E-04 | 0.005 | 20.250 |
| rs7973260 | 12 | 118375486 | G | A | -0.031 | 0.005 | 1.78E-09 | -1.16E-03 | 3.68E-04 | 0.002 | 38.440 |
| rs853679 | 6 | 28296863 | A | C | -0.023 | 0.005 | 6.62E-07 | -4.88E-04 | 3.95E-04 | 0.217 | 21.160 |
| rs9427622 | 1 | 196355524 | C | T | 0.021 | 0.004 | 4.75E-07 | 3.25E-04 | 2.98E-04 | 0.274 | 27.563 |

### Neuroticism || ieu-a-1007 on vegetarianism || ebi-a-GCST90096927 (8-SNP-P< 5.0x10^-8^)

| SNPID | chr | pos | effect_allele | other_allele | beta.exposure | se.exposure | *p*.exposure | beta.outcome | se.outcome | *p*.outcome | F |
| --- | --- | --- | --- | --- | --- | --- | --- | --- | --- | --- | --- |
| rs12903563 | 15 | 78033735 | C | T | -0.02 | 0.004 | 2.86E-08 | 3.20E-04 | 2.93E-04 | 0.274 | 25.000 |
| rs12938775 | 17 | 2574821 | A | G | -0.02 | 0.004 | 8.54E-09 | -5.94E-05 | 2.93E-04 | 0.839 | 25.000 |
| rs1557341 | 18 | 35127427 | C | A | -0.021 | 0.004 | 5.58E-09 | -2.36E-04 | 3.13E-04 | 0.451 | 27.563 |
| rs2572431 | 8 | 11105077 | T | C | 0.028 | 0.003 | 4.20E-16 | 6.05E-04 | 2.96E-04 | 0.041 | 87.111 |
| rs35688236 | 3 | 34582993 | C | A | -0.021 | 0.004 | 2.35E-08 | 3.16E-04 | 3.13E-04 | 0.314 | 27.563 |
| rs4938021 | 11 | 113364803 | T | C | 0.023 | 0.004 | 4.03E-10 | -2.63E-04 | 3.01E-04 | 0.383 | 33.063 |
| rs62057143 | 17 | 43901528 | C | T | 0.026 | 0.004 | 1.76E-10 | 2.65E-04 | 3.50E-04 | 0.449 | 42.250 |
| rs716804 | 11 | 10247295 | T | C | 0.02 | 0.003 | 3.82E-09 | 2.45E-05 | 2.93E-04 | 0.933 | 44.444 |

### Subjective well-being || ieu-a-1009 on vegetarianism || ebi-a-GCST90096927 (16-SNP-P< 5.0x10^-6^)

| SNPID | chr | pos | effect_allele | other_allele | beta.exposure | se.exposure | *p*.exposure | beta.outcome | se.outcome | *p*.outcome | F |
| --- | --- | --- | --- | --- | --- | --- | --- | --- | --- | --- | --- |
| rs10769190 | 11 | 46151490 | C | T | -0.018 | 0.004 | 2.19E-06 | 5.82E-04 | 3.59E-04 | 0.105 | 20.250 |
| rs11073619 | 15 | 85188839 | T | C | 0.025 | 0.005 | 3.42E-06 | 3.61E-04 | 4.88E-04 | 0.460 | 25.000 |
| rs11612312 | 12 | 52349088 | C | T | -0.019 | 0.004 | 1.37E-06 | 5.15E-04 | 3.65E-04 | 0.159 | 22.563 |
| rs12143280 | 1 | 211547522 | C | T | -0.038 | 0.008 | 6.46E-07 | -2.79E-04 | 6.12E-04 | 0.649 | 22.563 |
| rs12298541 | 12 | 66306441 | C | A | -0.016 | 0.003 | 2.28E-06 | -3.49E-04 | 3.07E-04 | 0.255 | 28.444 |
| rs13235506 | 7 | 53785701 | G | T | 0.034 | 0.007 | 1.96E-06 | -7.38E-05 | 6.51E-04 | 0.910 | 23.592 |
| rs17005492 | 4 | 140960772 | C | T | -0.018 | 0.004 | 2.01E-06 | 2.65E-05 | 3.53E-04 | 0.940 | 20.250 |
| rs17331012 | 4 | 12144655 | A | G | -0.023 | 0.005 | 1.10E-06 | 2.32E-04 | 4.39E-04 | 0.597 | 21.160 |
| rs17693963 | 6 | 27710165 | C | A | 0.026 | 0.005 | 9.25E-07 | 6.90E-05 | 4.44E-04 | 0.877 | 27.040 |
| rs2017279 | 10 | 3534725 | G | A | -0.016 | 0.003 | 3.15E-06 | -1.89E-04 | 3.17E-04 | 0.550 | 28.444 |
| rs2075677 | 20 | 47701024 | G | A | -0.021 | 0.004 | 1.88E-08 | 4.01E-04 | 3.39E-04 | 0.237 | 27.563 |
| rs258677 | 7 | 81733428 | T | G | 0.017 | 0.003 | 9.46E-08 | -5.24E-04 | 3.00E-04 | 0.080 | 32.111 |
| rs4589952 | 3 | 95702105 | T | C | -0.021 | 0.004 | 1.55E-06 | 3.18E-05 | 4.09E-04 | 0.938 | 27.563 |
| rs4842283 | 12 | 80822975 | A | C | -0.029 | 0.006 | 1.94E-07 | -2.51E-04 | 4.87E-04 | 0.607 | 23.361 |
| rs6587766 | 1 | 57708088 | T | C | 0.047 | 0.009 | 2.52E-07 | 6.51E-04 | 7.86E-04 | 0.407 | 27.272 |
| rs7149000 | 14 | 41897318 | G | A | 0.014 | 0.003 | 4.77E-06 | -5.69E-04 | 2.95E-04 | 0.054 | 21.778 |
